# Deletion of p75^NTR^ prevents vaso-obliteration and retinal neovascularization via activation of Trk- A receptor in ischemic retinopathy model

**DOI:** 10.1038/s41598-018-30029-0

**Published:** 2018-08-21

**Authors:** Sally L. Elshaer, Azza B. El-Remessy

**Affiliations:** 1 Augusta Biomedical Research Corporation, Augusta, GA 30912 USA; 2 Charlie Norwood VA Medical Center, Augusta, GA 30912 USA; 3 Ophthalmology Department, Hamilton Eye Institute, University of Tennessee Health Sciences Center, Memphis, TN 38163 USA

## Abstract

Ischemic retinopathy is characterized by ischemia followed by retinal neovascularization (RNV) resulting in visual impairment. Given the role of neuron-secreted growth factors in regulating angiogenesis, we examined how genetic deletion of the neurotrophin receptor; p75^NTR^ can overcome retinal ischemia using oxygen-induced retinopathy (OIR) mouse model. Wildtype (WT) or p75^NTR−/−^ mice pups were subjected to hyperoxia (70% O_2_, p7-p12) then returned to normal air (relative hypoxia, p12-p17). Vascular alterations were assessed at p12 and p17 time-points. Deletion of p75^NTR^ prevented hyperoxia-associated central vascular cell death (p12) and hypoxia-associated RNV and enhanced central vascular repair (p17). Decreased expression of apoptotic markers; preserved Akt survival signal decreased proNGF were also observed at p12. During hypoxia, deletion of p75^NTR^ maintained VEGF and VEGFR2 activation and restored NGF/proNGF and BDNF/proBDNF levels. Deletion of p75^NTR^ coincided with significant increases in expression and activation of NGF survival receptor, TrkA at basal and hyperoxic condition. Pharmacological inhibition of TrkA using compound K-252a (0.5 μg 1 μl^−1^/eye) resulted in 2-fold increase in pathological RNV and 1.34-fold increase in central vascular cell death in p75^NTR−/−^ pups. In conclusion, deletion of p75^NTR^ protected against retinal ischemia and prevented RNV, in part, through restoring neurotrophic support and activating TrkA receptor.

## Introduction

Ischemic retinopathy is a common characteristic of several ocular diseases including diabetic retinopathy, retinopathy of prematurity and retinal vein occlusion^1^. Ischemic retinopathy is characterized by an initial microvascular degeneration followed by a mal-adaptive pathological retinal neovascularization (RNV), consequent to hypoxia, in an attempt to reinstate metabolic equilibrium; that can result in visual impairment and eventually blindness^2^. Current therapeutic approaches including intravitreal injection of vascular endothelial growth factor (VEGF) neutralizing therapies are sight-saving, yet hindered by serious concerns such as late intervention, invasiveness and cost-prohibition^3^. Therefore, there is pressing need to develop new therapeutics that can preserve both neuronal and vascular function in ischemic retinopathy diseases.

Given that retina is a typical neurovascular unit, neurons and glial cells may interact with blood vessels to contribute to pathologic neovascularization by secreting growth factors and guidance cues^4–6^. Nerve growth factor (NGF) is well known for its role in regulating survival, growth and functional maintenance of neuronal cells as well as vasculature. In response to ischemic insult and hypoxia, NGF, among other angiogenic factors, will be released. Several studies showed that NGF mediates survival and angiogenic signal through activation of its high affinity; TrkA receptor^7–9^.

NGF is synthesized and released as precursor, proNGF that normally get cleaved to the mature form. We and others have shown that pro-oxidative milieu can impair maturation of NGF leading to accumulation of proNGF^10,11^. Contrast for mature form, proNGF has high affinity to neurotrophin death receptor; p75^NTR^, the first identified member of the tumor necrosis factor (TNF) receptor superfamily that have intracellular death domain^12^. Several reports demonstrated the action of proNGF, mediating apoptosis via activating p75^NTR^ ^13–15^ and forming a co-receptor with sortilin; a member of Vps10p-domain receptor family^16^. Nevertheless, the role of proNGF in regulating cell survival and reparative angiogenesis is ill-defined. Few reports showed that proNGF-mediated angiogenic behavior in breast cancer cells^17^ and retinal endothelial cells^18^ that was regulated through activation of TrkA but not p75^NTR^. In the latter study, our group observed that inhibition of p75^NTR^ was associated with enhanced TrkA activation^18^, suggesting a cross-talk between p75^NTR^ expression and TrkA activation. Despite these significant findings, there is gap in knowledge about the interplay between NGF and proNGF and their receptors p75^NTR^ and TrkA in response to hypoxia and angiogenesis.

In the current study using oxygen-induced retinopathy (OIR) mouse model, a standard model for ischemic retinopathy^19^, we examined the impact of genetic deletion of p75^NTR^ on vascular cell death as well as pathological neovascularization and reparative angiogenesis. Here, we share the findings that genetic deletion of p75^NTR^ prevented vascular cell death, enhanced central reparative capillary growth and prevented pathological neovascularization in mouse retina. The mechanisms involve restoring levels of NGF and BDNF, preserving VEGF signaling and enhancing TrkA-mediated survival and angiogenic signal. Therefore, we postulate that development of inhibitors against p75^NTR^ receptor can provide potential alternative therapeutic strategy for ischemic retinopathy.

## Materials and Methods

### Animals

All animal experiments were performed in accordance with relevant guidelines and regulations of “Association for Research in Vision and Ophthalmology” statement for use of animals in ophthalmic and vision research, and were approved by the “Charlie Norwood VA Medical Center Animal Care and Use Committee”, ACORP#16–01–088. The p75^NTR^, B6.129S4Ngfr^tm1Jae^/J (p75^NTR+/−^, exon III knockout mice^20^) were obtained from Jackson Laboratories (Bar Harbor, Maine, USA) and crossed with C57BL6/J mice (Jackson Laboratories). The WT and p75^NTR+/−^ mice were crossed then back-crossed again to establish a colony of homozygous p75^NTR−/−^ and WT breeders that produced the mice used in the current study.

### Oxygen-induced retinopathy (OIR) mouse model

OIR model was performed as previously described by Smith *et al*.^21^. Briefly, on postnatal day 7 (p7), WT and p75^NTR−/−^ mice pups were placed along with their dams into a custom-built chamber (Biospherix, Redfield, NY) in which the partial pressure of oxygen was maintained at 70% for 5 days (hyperoxic stage). Pups were then returned to room air (21% oxygen, hypoxic stage) for 5 days (p12-p17). Central capillary drop-out (CDO) and retinal neovascularization (RNV) in OIR-exposed mice pups were performed at p12 and p17 time points, respectively, as described previously^21,22^ whereas, biochemical assays; Western blotting and PCR were performed at p12 and p14 time points. At the selected time points, mice pups were euthanized in carbon-dioxide chamber (2% flow rate for 5 min) followed by cervical dislocation. One eye was enucleated and fixed in 4% paraformaldehyde overnight to be flat-mounted for morphological studies. For the other eye, retinas were isolated and snap frozen for further biochemical analysis.

### Intravitreal injection

Mice were anesthetized by intraperitoneal injection of ketamine (100 mg/kg) xylazine (10 mg/kg) mixture and complete anesthesia was confirmed by loss of reflex to sharp paw pinch. K252a (Sigma, MO, USA; 0.5 μg μL^−1^/eye) or Dimethyl sulphoxide (DMSO) were injected intravitreally at p12 using a Hamilton syringe with 32-gauge glass capillary. Pups were left to recover (6-hours) from exposure to hyperoxia before intravitreal injection.

### Morphological studies

Retinas were dissected and permealized for 15 minutes with 0.3% Triton X-PBS then stained overnight at 4 °C with isolectin; biotinylated griffonia (bandeiraea) simplicifolia lectin I (GSL I, BSL I), (Vector Labs, CA, USA; 1% in 5% normal goat serum in 0.3% Triton X-PBS) followed by incubation with secondary antibody; Texas red® avidin D (Vector labs, CA, USA; 0.5% in 5% normal goat serum in 0.3% Triton X-PBS). Retinal perfusion was assessed by intraperitoneal injection of FITC-dextran (Mol. WT. 2,000,000, Sigma, 50 mg mL^−1^, 100 μL/pup) 30 minutes prior to sacrifice as described previously^23^. Lectin-stained and/or FITC-perfused retinas were flat-mounted onto Super-frost/Plus microscope slides (Fisher Scientific, MA, USA) with the photoreceptor side facing down and imbedded in Vectashield mounting media for fluorescence (Vector Labs, CA, USA). Slides were photo-micrographed at 5X using a Zeiss AxioObserver.Z1. Images were assembled into a single file using photoshop software (CS6, Adobe system incorporated). CDO and RNV were quantified as previously described^24^.

### Western blot analysis

Frozen retinas were placed into protein lysis buffer (Millipore) and briefly homogenized. Retinal lysates were centrifuged and 35–50 μg were resolved on an SDS-PAGE gel (4–20% gradient Tris glycine precast gel, BioRad) and electro-blotted to nitrocellulose membranes. Membranes were blocked with 5% milk or BSA in PBS-tween and incubated overnight in 4 °C with the following primary antibodies: p75^NTR^ (kind gift from Dr. Bruce Carter, Department of Biochemistry, Vanderbilt University), Akt (#9272, Cell Signaling), p-Akt (#9275 S, Cell Signaling), cleaved-PARP (#5625, Cell Signaling), total PARP (#9532, Cell Signaling), cleaved caspase-3 (#9664, Cell Signaling), TrkA (#76291, Abcam), phospho-TrkA (#1445, Abcam), NGF (# AN-240, Alomone), proNGF (#ANT-005, Alomone), sortilin (#16640, Abcam), BDNF and proBDNF (SC-546, Santa Cruz), VEGF (#ABS82, Millipore), VEGFR2 (#2472, Cell Signaling), phopho-VEGFR2 (#2474, Cell Signaling), then re-probed with the primary antibodies for the house-keeping proteins; actin (#A5441, Sigma) or tubulin (#ab4074, Abcam) to confirm equal loading. Membranes were incubated with horseradish peroxidase (HRP)-conjugated anti-mouse or HRP-conjugated anti-rabbit secondary antibodies (Millipore) for 2 h at room temperature. The films were scanned with FluorChem™ FC3 (protein simple) and the band intensity was quantified using densitometry software version (Fiji) and expressed as a relative optical density (OD).

### Quantitative real-time (RT-PCR)

Retinas samples were processed using (mirVANA^TM^ PARIS^TM^ Kit) and RNA was purified and quantified as described previously^25^ following the manufacturer’s instructions. A one-step quantitative RT-PCR kit (Invitrogen) was used to amplify 10 ng retinal mRNA as described previously^26,27^. PCR primers (listed in Table 1) were obtained from Integrated DNA Technologies (Coralville, IA, USA). Quantitative PCR was conducted using StepOnePlus qPCR system (Applied BioSystems, Life Technologies). The percent expression of various genes was normalized to 18S and expressed relative to WT normoxic controls.

**Table 1:** For primer sequences.

Gene-mouse	Forward primer	Reverse primer
TrkA	5′-TGGGCAGAGAATGATGTGGG-3′	5′-CGAGAAGGGGATGCACCAAT-3′
VEGF	5′-CACGACAGAAGGAGAGCAGAA-3′	5′-CGCTGGTAGACGTCCATG-3′
NGF	5′-AGGCCCATGGTACAATCCCTTTCA-3′	5′-ATCTCCAACCCACACACTGACACT-3′
18S	5′-CGCGGTTCTATTTTGTTGGT-3′	5′-AGTCGGCATCGTTTATGGTC-3′

### Statistical analysis

All the data are expressed as mean ± SEM. Differences between 2 groups for morphological studies were detected using un-paired Student T-test. Two-way ANOVA was used to assess interaction between two variables; genetic background (WT vs. p75^NTR−/−^) and OIR exposure (normoxia vs. hyperoxia/hypoxia). Tukey-Kramer post-multiple comparisons was used for significant interactions among various groups. Significance for all tests was determined at α = 0.05, Graphpad Prism, Ver.6.

## Results

### Hyperoxia but not hypoxia triggered p75^NTR^ expression in oxygen-induced retinopathy (OIR)

**Figure 1:**
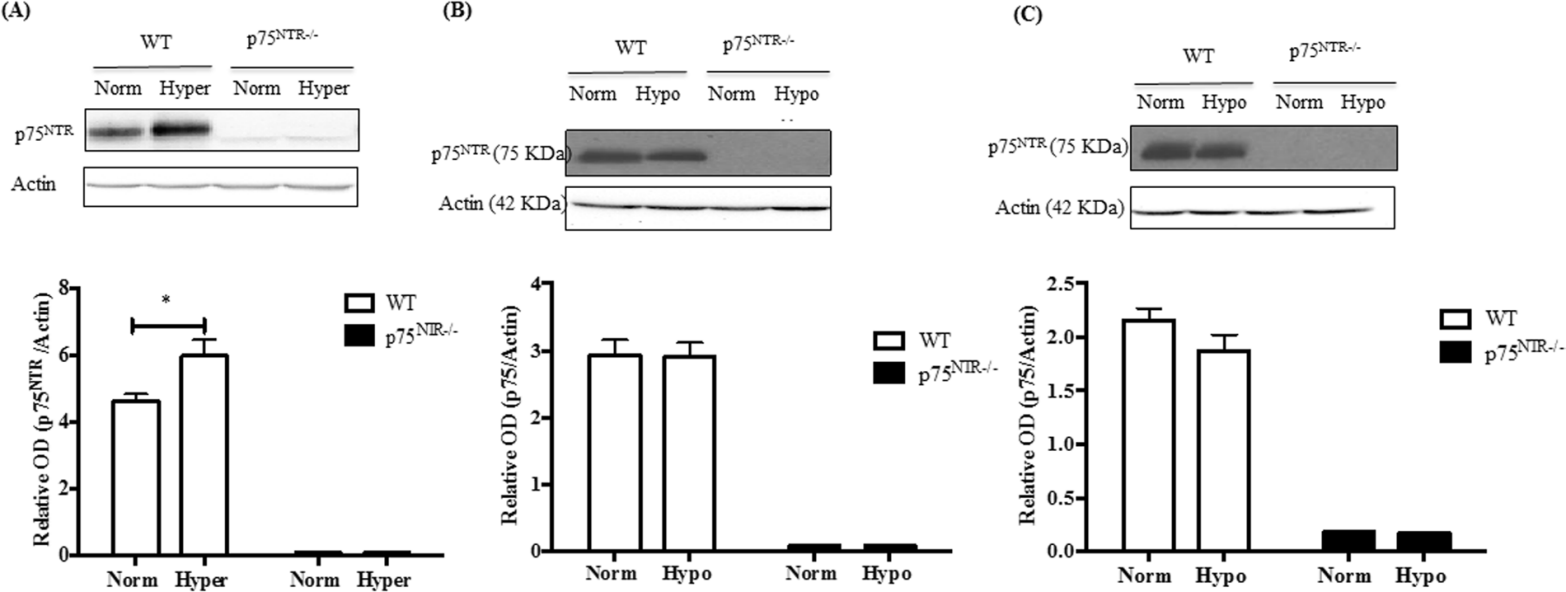
Hyperoxia, but not hypoxia triggered p75^NTR^ expression in WT-pups in OIR model. (**A**) Exposing WT pups to 70% oxygen from p7-p12 significantly increased p75^NTR^ expression in WT-pups (*significant compared to WT-controls using two-way ANOVA, p < 0.05, n = 3–4). (**B**) Western blotting representative and bar graph of p75^NTR^ receptor expression in WT pups by p14 showing no alteration during hypoxic stage of OIR (n = 4–6). (**C**) Western blotting representative and bar graph of p75^NTR^ receptor expression in WT pups by p17 showing a trend for decreased p75^NTR^ expression that was not statistically significant (n = 4).

We aimed to examine the vascular protective effects of targeting p75^NTR^ in ischemic retinopathy using p75^NTR−/−^, exon III knockout mice^20^. OIR is a commonly used model to study ischemic retinopathy as it has two distinctive stages; capillary dropout and ischemia at p12 followed by retinal neovascularization at p17. At postnatal day-7, mice pups were placed in partial pressure of oxygen at 70% for 5 days (hyperoxic stage, p7-p12). At p12, pups were returned to room air (21% oxygen, relative hypoxic stage) for 5 days (hypoxic stage, p12-p17). Exposing the developing retina of WT mice to hyperoxia resulted in significant (1.4-fold) increase in p75^NTR^ expression compared to normoxic controls (Fig. 1A). Next, we examined the expression of p75^NTR^ receptor in WT mice during the hypoxic stage of OIR (p12-p17). As shown in Fig. 1B,C, there was no significant difference in p75^NTR^ expression at p14 or p17 time points. Moreover, we assessed expression of sortilin receptor, a member of Vps10p-domain receptor family, known to be co-receptor for neurotrophin signaling^16^. As shown in Supplementary Fig. 1, sortilin expression was not affected during hyperoxia at p12 (Supplementary Fig. 1A), or during relative hypoxia at p14 (Supplementary Fig. 1B) in WT pups. In addition, genetic deletion of p75^NTR−/−^ receptor did not impact expression of sortilin under normoxia, during hyperoxia (p7-p12) or during hypoxia (p12-p17).

### Deletion of p75^NTR^ attenuated hyperoxia-increase in proNGF and decrease in NGF

**Figure 2:**
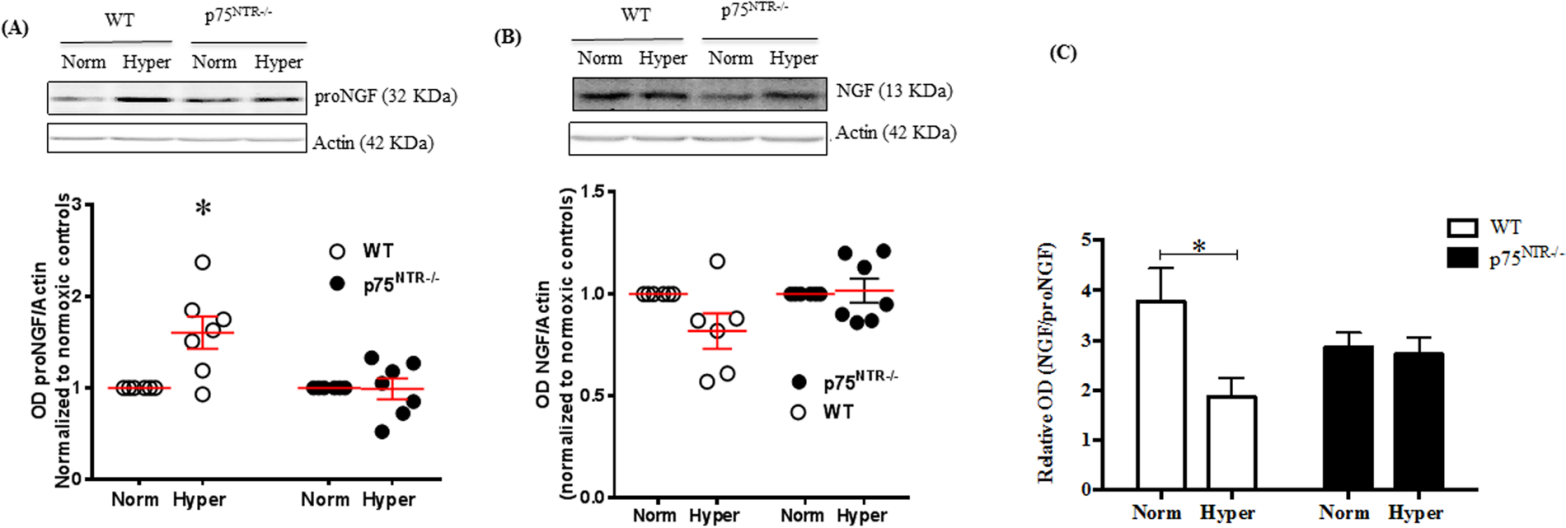
Deletion of p75^NTR^ attenuated hyperoxia-induced increase in proNGF and decrease in NGF. (**A**) Representative Western blotting and scatter graph showing significant increase in proNGF protein levels in retinas from WT, but not in p75^NTR−/−^ in response to hyperoxia. (*significant compared to the rest of the groups using two-way ANOVA, p < 0.05, n = 6–8). (**B**) Representative Western blotting and scatter graph showing a tendency of NGF protein level to be decreased in WT (P = 0.07), but preserved-level in p75^NTR−/−^ during hyperoxia. (**C**) Bar graph showing significant decrease in of NGF/proNGF ratio in retinas from WT, but not p75^NTR−/−^ pups in response to hyperoxia (*significant compared to WT normoxic controls, using two-way ANOVA, p < 0.05, n = 6–8).

We have previously shown that, exposure to hyperglycemia disturbed homeostasis of NGF by accumulation of its precursor; proNGF at the expense of the mature form; NGF^11,28^. Similar observation was noticed in OIR-exposed WT pups, where, early hyperoxic stage resulted in significant (1.7-fold) increase in proNGF expression (Fig. 2A) whereas; NGF showed mild decrease (Fig. 2B) compared to normoxic controls. Imbalance in both proNGF and NGF was ameliorated with genetic deletion of p75^NTR^ (Fig. 2A,B). Blotting the ratio of NGF/proNGF showed 50% decrease in WT pups after exposure to hyperoxia while the ratio was preserved in p75^NTR−/−^ retinas, as shown in Fig. 2C. These results suggest that hyperoxia shifted the homeostasis of NGF signaling towards apoptotic pathway of proNGF/p75^NTR^ at the expense of survival pathway; NGF signaling pathway.

### Deletion of p75^NTR^ preserved survival and attenuated hyperoxia-mediated apoptosis

**Figure 3:**
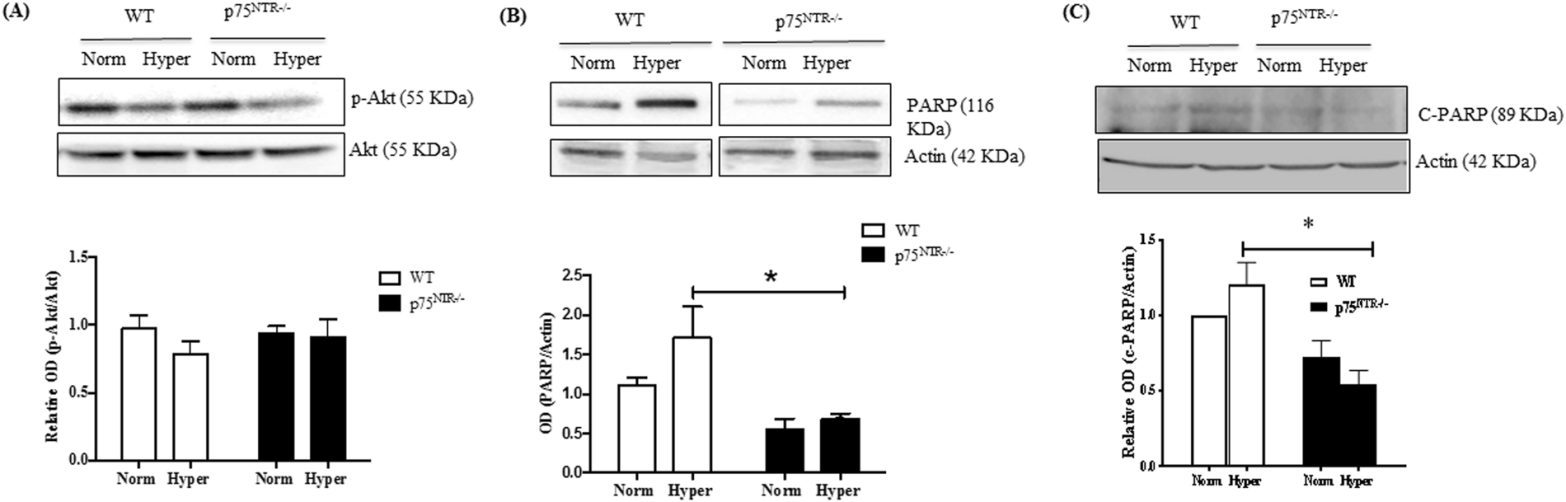
Deletion of p75^NTR^ preserved survival signal and attenuated hyperoxia-mediated apoptosis. (**A**) Representative Western blotting and bar graph showing modest decrease in Akt activation (Y308) in response to hyperoxia at p12 retinal lysates of WT and p75^NTR−/−^ mice (n = 5–7). (**B**) Representative Western blotting and bar graph of total PARP expression in p12 retinal lysates of WT and p75^NTR−/−^ mice (*significant using two-way ANOVA, p < 0.05, n = 7). (**C**) Representative Western blotting and bar graph of cleaved PARP expression in p12 retinal lysates of WT and p75^NTR−/−^ mice (*significant using two-way ANOVA, p < 0.05, n = 4–6).

Exposure to hyperoxia tended to decrease the activation of the survival kinase; Akt by 30% in retinas of WT pups, but was preserved in p75^NTR−/−^ mice (Fig. 3A). Hyperoxia resulted in increased total and cleaved PARP in retinas from WT pups (Fig. 3B,C). Furthermore, retinas from p75^NTR−/−^ pups showed ~50% lower levels of both total-PARP (Fig. 3B) and cleaved-PARP (Fig. 3C) compared to their WT littermates either at normoxic or hyperoxic conditions.

### Deletion of p75^NTR^ attenuated hyperoxia-induced capillary dropout at p12

**Figure 4:**
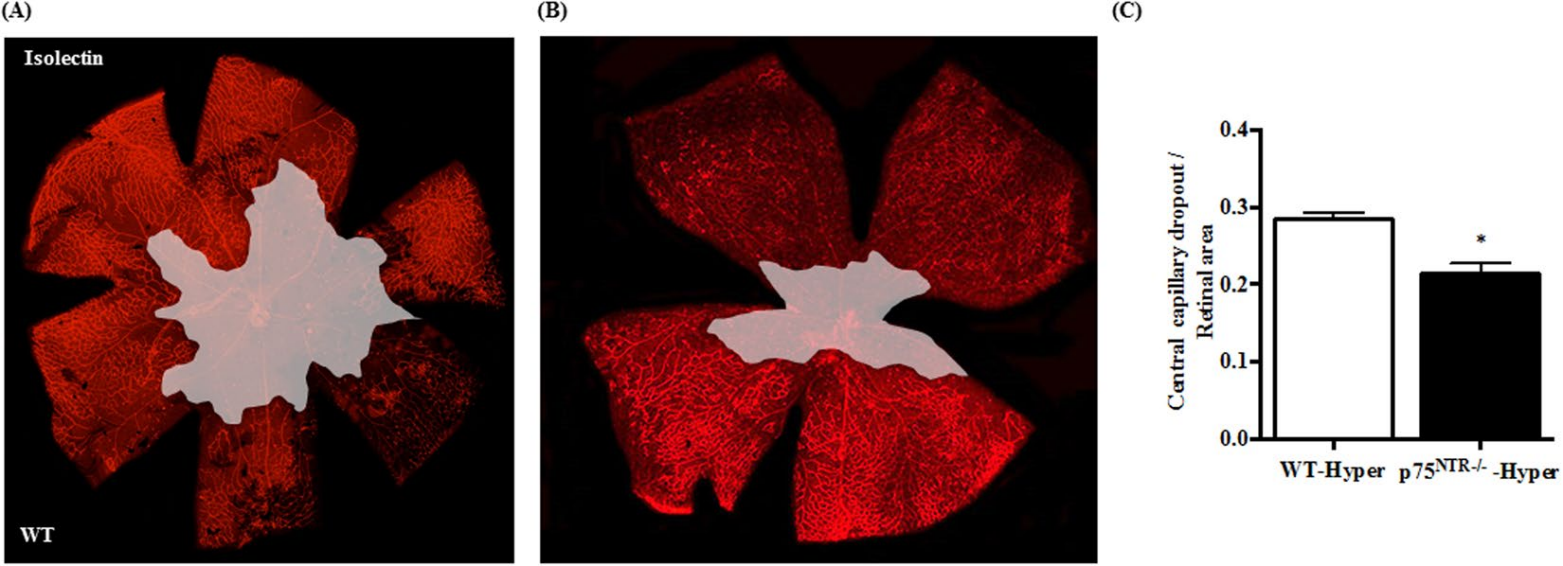
Deletion of p75^NTR^ attenuated hyperoxia-induced retinal central vaso-obliteration. (**A**,**B**) Representative images of Isolectin GS-stained retinal flat mounts of WT and p75^NTR−/−^ pups showing capillary drop-out (CDO) illustrated by encircled area at the center at p12 (magnification is 5X). (**C**) Bar graph and statistical analysis using unpaired student T-test for CDO showing significant reduction of CDO in p75^NTR−/−^ compared to WT pups in response to hyperoxia (*significant compared to WT-hyperoxic group, p < 0.05, n = 8–12). (Hyper) hyperoxia-exposed group, (Norm) normoxic controls, (CDO): central capillary dropout.

By p12, retinal ischemia and vaso-obliteration evident by central capillary dropout (CDO) reach its maximum in OIR model. Indeed, hyperoxia caused marked vaso-obliteration and CDO, illustrated by the central encircled area (Fig. 4A) in retinas from WT pups but not in p75^NTR−/−^ pups (Fig. 4B). Statistical analysis showed significant reduction by 34% in CDO in p75^NTR−/−^ pups compared to their WT littermates (Fig. 4C). These vascular manifestations coincided with increased expression of proNGF, p75^NTR^ and apoptotic markers in WT pups but not p75^NTR−/−^ during hyperoxia.

### Relative hypoxia triggers retinal neovascularization in WT but not p75^NTR−/−^ at p17

**Figure 5:**
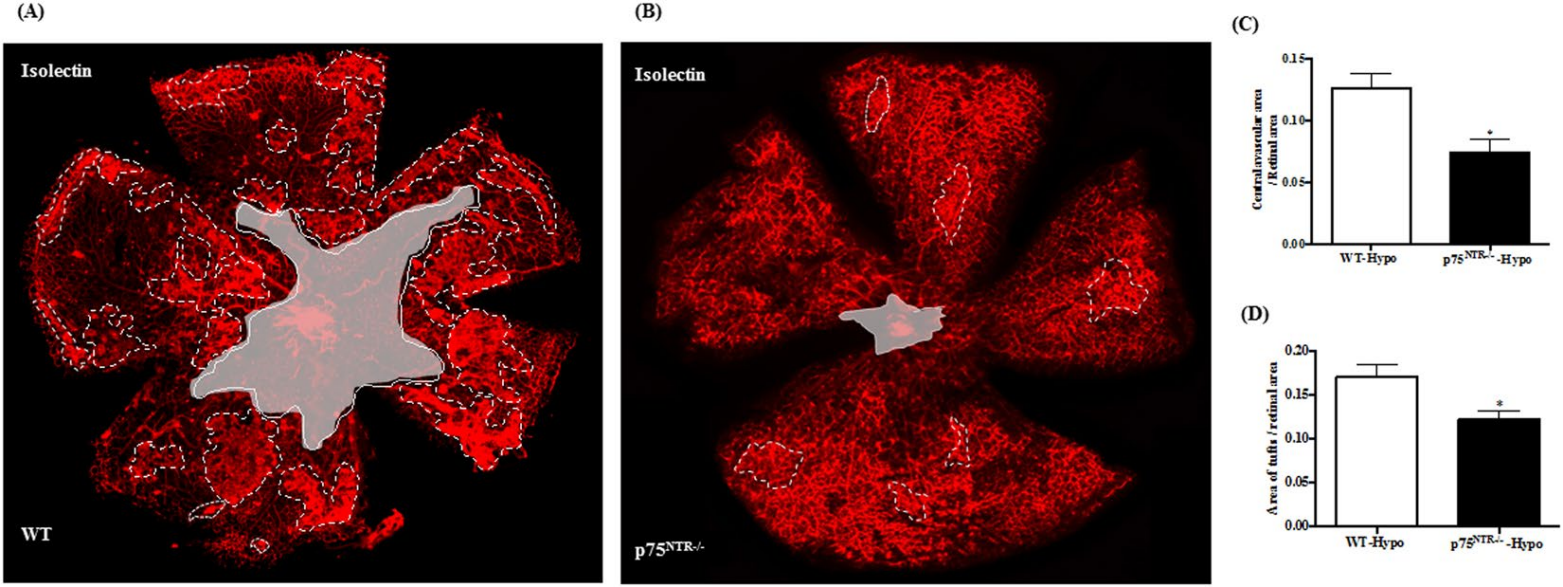
Deletion of p75^NTR^ attenuated hypoxia-induced neovascularization and enhances reparative angiogenesis. (**A**,**B**) Representative images of GS-isolectin stained retinal flat mounts of WT and p75^NTR−/−^ pups at p17, showing pathological retinal neovascularization (RNV, encircled by dotted white line) and central avascular area (encircled area at the center). Images were taken on 5X magnification. Bar graph of RNV (**C**) and central avascular area (**D**) showing significant decrease in both manifestations in p75^NTR−/−^ pups by p14 during hypoxic stage of OIR (*significant using unpaired student T-test, p < 0.05, n = 7–14). Hypo: relative hypoxia-exposed groups, RNV: retinal neovascularization.

At p12, pups that were exposed to hyperoxia were returned to room air (21% oxygen, relative hypoxic stage) for 5 days (p12-p17). Relative hypoxia triggered significant pathological retinal neovascularization in the mid-periphery of WT retinas, growing towards the vitreous (encircled in Fig. 5A by dotted white line). Additionally, relative hypoxia impaired intra-retinal reparative angiogenesis towards the central avascular area in WT-pups (Fig. 5A, encircled area at the center). Deletion of p75^NTR^ receptor significantly attenuated hypoxia-induced pathological RNV by 42% compared to WT-pups (Fig. 5B,C). More importantly, Deletion of p75^NTR^ stimulated reparative angiogenesis as indicated by growth of capillaries toward the central retina and decreasing central avascular area by ~32% compared to WT-pups during hypoxic stage of OIR model (Fig. 5B,D).

### Hypoxia triggered VEGF expression and VEGFR2 activation in both WT and p75^NTR^ knockout

**Figure 6:**
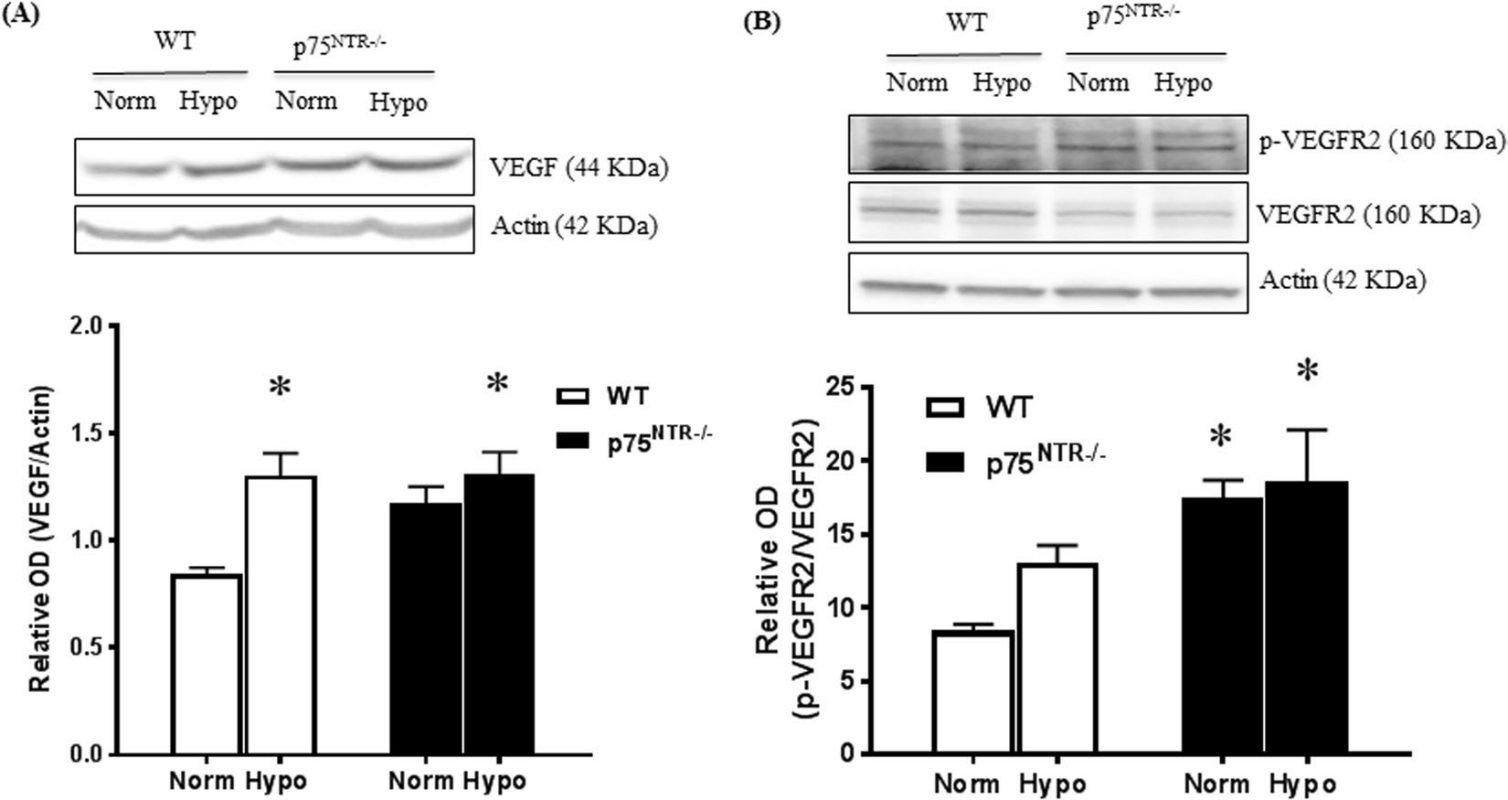
Deletion of p75^NTR^ preserved hypoxia-induced VEGF expression and VEGFR2 activation. (**A**) Representative Western blotting and bar graph of retinal VEGF protein expression from WT and p75^NTR−/−^ pups exposed to OIR at p14. Two-way ANOVA showed significant effect of hypoxia increasing VEGF both in WT and p75^NTR−/−^ pups (*significant compared to WT normoxic group, p < 0.05, n = 6–12). (**B**) Representative Western blotting and bar graph of pVEGFR2 compared to its total protein VEGFR2 level. Two-way ANOVA showed marked gene deletion effect where p75^NTR−/−^ showed significant increase in pVEGFR2 under normoxia or hypoxia compared to WT-normoxic controls (*significant compared to WT normoxic group using, p < 0.05, n = 4–5).

Expression of vascular endothelial growth factor (VEGF), a well-studied angiogenic factor in OIR model is triggered by hypoxia and correlates with RNV^29^. Therefore, we investigated impact of deleting p75^NTR^ on VEGF level and its signal. At p14, hypoxia resulted in significant 2.2-fold increase in VEGF mRNA expression in WT pups (Supplementary Fig. 2A) and 1.53-fold increase in VEGF protein expression in retinas of WT pups as compared to normoxic controls (Fig. 6A). Deletion of p75^NTR^ receptor tended to alter but did not reach statistical significance the basal expression of VEGF mRNA or protein in normoxia. Yet upon hypoxia exposure, retinas from p75^NTR−/−^ pups showed tendency of increased mRNA expression of VEGF that was not statistically significant (Supplementary Fig. 2A), and significant increase by 1.6-fold in VEGF protein as compared to normoxic controls (Fig. 6A). Next, we examined activation of VEGF receptor-2 (VEGFR2) known to mediate angiogenic signal. Exposure to relative hypoxia resulted in an increase in VEGFR2 activation that did not reach statistical analysis in retinas from WT pups compared to normoxic controls (Fig. 6B). Interestingly, retinas from p75^NTR−/−^ pups exhibited significant activation of VEGFR2 at both basal and hypoxic conditions compared to WT-normoxic controls (Fig. 6B).

### Deletion of p75^NTR^ attenuated hypoxia-induced increase in proNGF and preserved NGF/proNGF ratio

**Figure 7:**
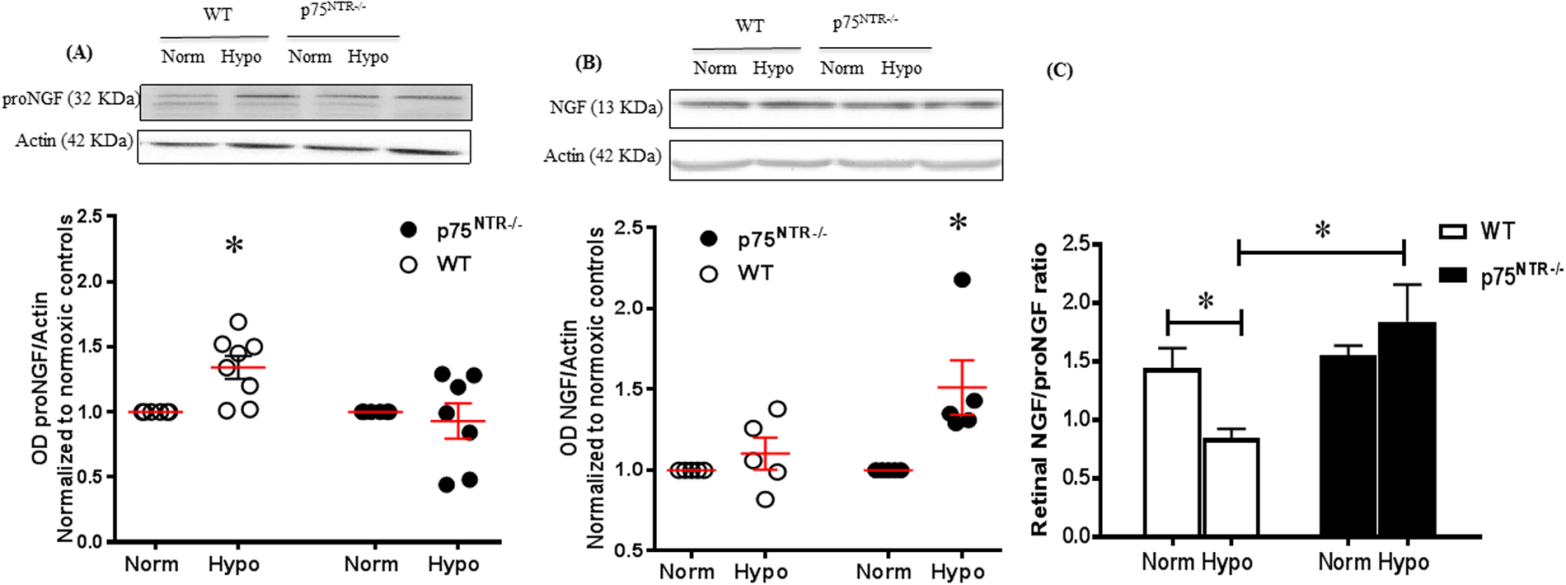
Deletion of p75^NTR^ attenuated hypoxia-induced increase in proNGF and restored ratio of NGF/proNGF. (**A**) Representative Western blotting and scatter graph for proNGF showing that hypoxia caused significant increase in proNGF levels in retinas from WT-pups but not p75^NTR−/−^ compared to normoxic control at p14. (**B**) Representative Western blotting and scatter graph for NGF showing that hypoxia caused significant increase in NGF level in retinas from p75^NTR−/−^, but not WT compared to their normoxic controls. (**C**) Bar graph of NGF/proNGF ratio and Two-way ANOVA statistical analysis showing significant decrease in retinal NGF/proNGF ratio in WT-pups during hypoxia while p75^NTR−/−^ pups maintained balanced ratio of NGF/proNGF (*significant using Two-way ANOVA, p < 0.05, n = 5–7).

At p14, relative hypoxia caused significant (1.45-fold) increase of proNGF in retinas from WT-pups compared to normoxic controls (Fig. 7A). In contrast, hypoxia did not significantly alter NGF levels in WT-pups compared to normoxic controls (Fig. 7B). Deletion of p75^NTR^ prevented hypoxia-induced increase in retinal proNGF level and significantly increased NGF levels (1.4-fold) compared to p75^NTR−/−^ and WT normoxic controls (Fig. 7A,B). Next, we investigated impact of hypoxia and p75^NTR^ deletion on mRNA expression of NGF. As shown in Supplementary Fig. 2B, exposure to hypoxia significantly decreased NGF expression in retinas from WT-mice. In contrast, p75^NTR^ gene deletion did not alter expression of NGF under normoxia or hypoxia compared to WT-normoxic controls. These results suggest that the observed increase of proNGF in response to hypoxia and the increase in NGF levels associated with p75^NTR^ deletion are post-transcriptional. Blotting the ratio of retinal NGF/proNGF protein showed that hypoxia caused significant 40% decrease in NGF/proNGF in retinas from WT pups compared to normoxic controls but not in p75^NTR−/−^ pups (Fig. 7C). Similar results showing that hypoxia altered the levels and ratio between the precursor and mature form of the brain derived neurotrophic factor (BDNF) were observed. As shown in Supplementary Fig. 3, hypoxia triggered significant increase in retinal proBDNF level in WT-pups but not in p75^NTR−/−^. In response to hypoxia, the ratio of BDNF/proBDNF was significantly decreased in WT pups but not in p75^NTR−/−^.

### Deletion of p75^NTR^ enhanced the NGF survival receptor TrkA expression and activation at p12

**Figure 8:**
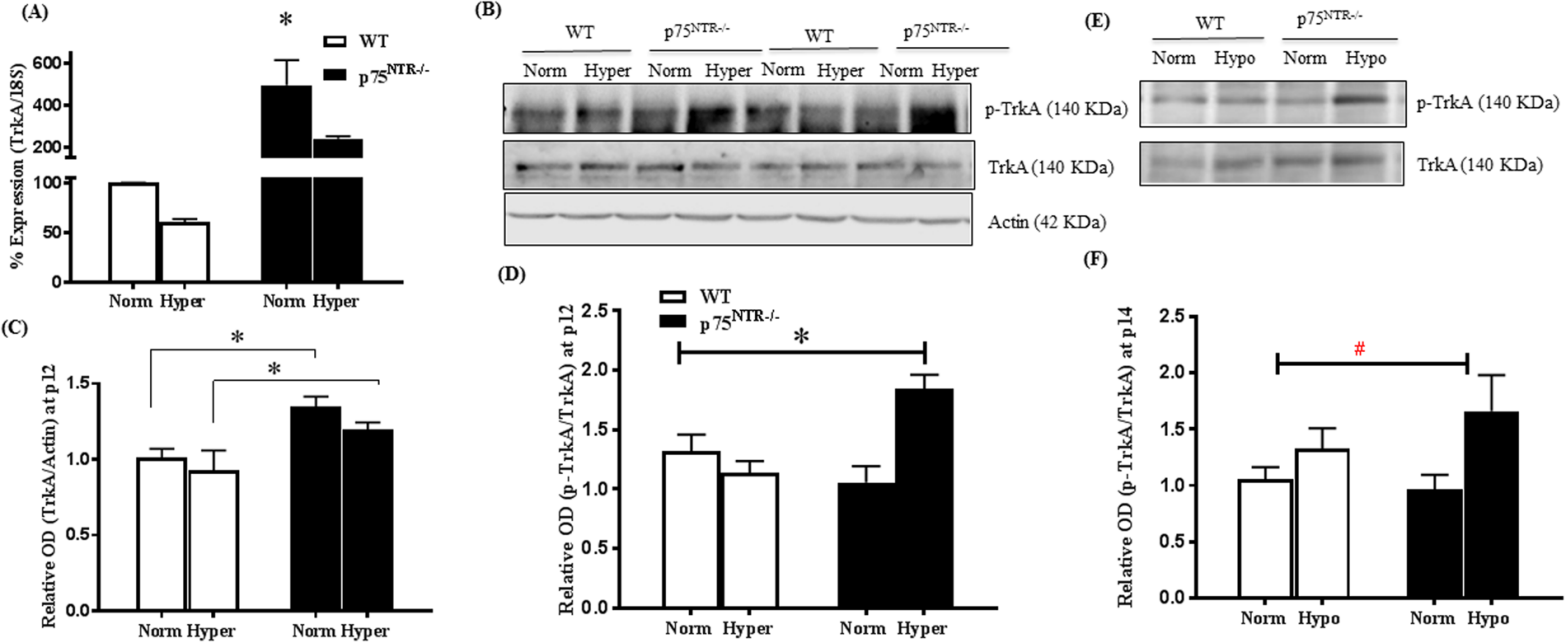
Deletion of p75^NTR^ enhanced expression and activation of NGF survival receptor; TrkA. (**A**) Quantitative real-time PCR of TrkA gene expression in p12 WT and p75^NTR−/−^ exposed to OIR showing significant increase in basal mRNA expression in p75^NTR−/−^ retinas compared to the other groups (*significant using Two-Way ANOVA, p < 0.05, n = 6). (**B**,**C**) Representative Western blotting and bar graph statistical analysis of TrkA expression by p12 showing significant gene effect and significant increase in TrkA expression in p75^NTR−/−^ pups compared to their corresponding WT-controls under normoxia and hyperoxia (*significant using Two-way ANOVA, p < 0.05, n = 6–9). (**B**,**D**) Representative Western blotting and bar graph statistical analysis of pTrkA activation by p12 showing an overall significant gene effect and a significant increase in pTrkA activation (Y490) in p75^NTR−/−^ pups exposed to hyperoxia compared to the rest of the groups (*significant using Two-way ANOVA, p < 0.05, n = 6–9). (**E**,**F**) Representative Western blotting and bar graph statistical analysis of pTrkA activation by p14 showing an overall significant effect of hypoxia (**#**) to increase activation of pTrkA (Y490) in p75NTR^−/−^ pups, however there was no significant difference between individual groups (n = 6–9).

TrkA is a tyrosine kinase receptor that selectively binds NGF to mediate survival and angiogenic signal (reviewed in^15^). We assessed the expression and activation pattern of TrkA during the two stages of OIR. Two-way ANOVA analysis showed significant effect of hyperoxia where it decreased mRNA expression of TrkA by ~40% in retinas from WT-pups and 50% from p75^NTR−/−^ compared to corresponding normoxic controls (Fig. 8A). Two-way ANOVA analysis showed significant gene effect where deletion of p75^NTR^ receptor significantly increased mRNA expression of retinal TrkA by ~5-fold compared to WT-pups at normoxia and 2.5-fold increase upon hyperoxia compared to WT retinas (Fig. 8A). Next, we evaluated protein expression of TrkA receptor during hyperoxic stage of OIR (Fig. 8B,C). Two-way ANOVA analysis showed significant effect of deleting p75^NTR^ gene where retinas from p75^NTR−/−^ pups showed significant increase in TrkA protein level compared to WT under normoxia and hyperoxia (Fig. 8B,C). Remarkably, Two-way ANOVA analysis showed significant effect of hyperoxia on TrkA activation. As shown in Fig. 8B,D, retinas from p75^NTR−/−^ pups exposed to hyperoxia showed significant TrkA activation compared to p75^NTR−/−^ normoxic controls and to retinas from WT-pups exposed to normoxia and hyperoxia. These results of increased TrkA expression and activation in p75^NTR−/−^ coincided with improved NGF/proNGF and vaso-protective effects observed at the ischemic stage of OIR at p12. Next, we examined impact of hypoxia; the second stage of OIR and p75^NTR^ deletion on TrkA expression and activation. At p14, there was no significant impact of relative hypoxia or gene deletion on TrkA receptor mRNA or protein expression compared to normoxic controls (Supplementary Fig. 4). Interestingly, Two-way ANOVA analysis showed a significant impact of hypoxia on p-TrkA (Y490) activation where retinas from both WT and p75^NTR^ hypoxic pups tended to show higher TrkA activation (1.4-fold and 1.7-fold) compared to their normoxic controls but it did not reach statistical significance (Fig. 8E,F). These results indicate that genetic deletion of p75^NTR^ exerted vascular protective effect via preserving and enhancing TrkA cell survival signal during hyperoxic-ischemic phase more than angiogenic signal during hypoxic-angiogenic phase.

### TrkA activity is required for the vaso-protective effects observed in p75^NTR−/−^ but not WT

**Figure 9:**
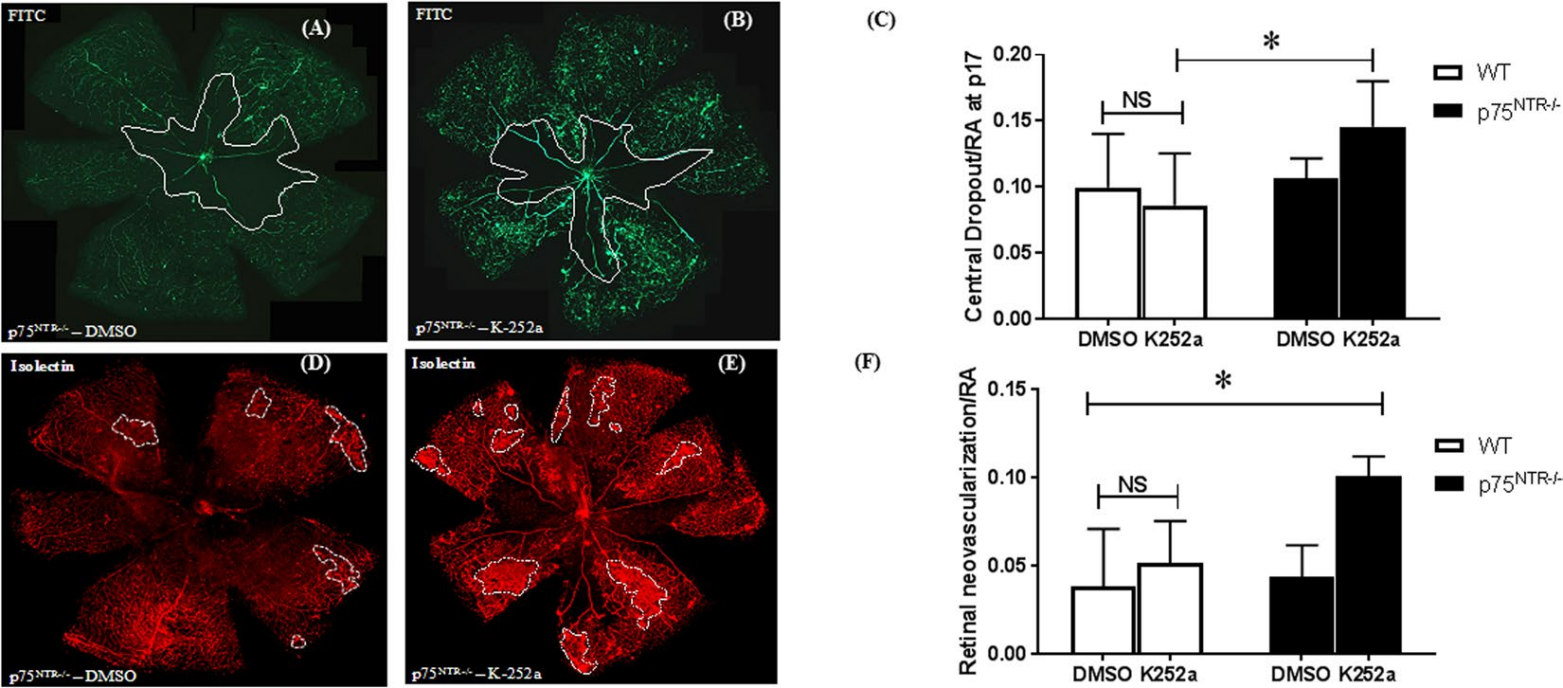
TrkA activity is required for the vaso-protective effects observed in p75^NTR−/−^ pups. **(A**,**B)** Representatives of p17 retinal flat mounts of FITC-perfused p75^NTR−/−^ pups exposed to OIR and receiving DMSO or K252a (0.5 μg μL^−1^/eye) showing central avascular area encircled in the center by white line, 5X magnification. **(C)** Statistical analysis of central capillary dropout (CDO) area showing significant increase of CDO in p75^NTR−/−^ pups that received intravitreal injection of K252a compared to DMSO-receiving p75^NTR−/−^ group or WT-pups that received K252a. There was no significant difference between WT-pups that received K252a compared to DMSO injection. (*Significant using Two-Way ANOVA, p < 0.05, n = 5–7). (**D**,**E**) Representatives of the same p75^NTR−/−^ retinal flat mounts stained with GS-isolectin to illustrate retinal neovascularization (RNV) at retinal mid-periphery (encircled by dotted white line, 5X magnification). (**F**) Statistical analysis using two-way ANOVA showing significant increase of RNV in p75^NTR−/−^ pups that received intravitreal injection of K252a compared to DMSO-injected WT or p75^NTR−/−^ groups. There was no significant difference between WT-pups that received K252a compared to DMSO injection. (*Significant using Two-Way ANOVA, p < 0.05, n = 4–7).

To examine the specific contribution of TrkA activation in vascular protection observed in p75^NTR−/−^ mice, we used compound K-252a, a staurosporine analog that is potent inhibitor of various Trk receptors^30^. K-252a was dissolved in dimethyl sulfoxide (DMSO) and administered via intra-vitreal route (0.5 μg μl^−1^/eye) at p12. Animals were perfused with FITC-dextran 30 minutes before sacrifice to examine capillary perfusion and central avascular area (Fig. 9A,B, Supplementary Fig. 5A,B) and after sacrifice at p17, retinas were flat-mounted and stained with Isolectin-GS to examine RNV (Fig. 9D,E, Supplementary Fig. 5C-D). WT data showed that treatment with K-252a did not significantly alter central avascular area (Supplementary Fig. 5A,B) or RNV (Supplementary Fig. 5C,D) compared to DMSO-injected controls. In contrast, injection of K-252a into retinas of p75^NTR−/−^ pups resulted in significant 1.34-fold increase in central avascular area (Fig. 9B,C) and 2-fold increase in RNV (Fig. 9E,F) when compared to DMSO-injected controls.

## Discussion

Retinal ischemia and the subsequent secretion of angiogenic factors including VEGF and NGF are thought to be common precursors to retinal neovascularization. While targeting VEGF has proven effective clinically in preventing macular edema and RNV, it deprives the retina from neuroprotective factors^31^. Therefore, the challenge remains to identify strategies to vascularize the ischemic retina and to maintain homeostasis of retinal function as a neurovascular unit. The findings from this study support targeting p75^NTR^ as a novel and effective vascular protective strategy for ischemic retinopathy as follows: **A**. Deletion of the p75^NTR^ reduced hyperoxia-mediated vaso-obliteration, hypoxia-induced pathological neovascularization and enhanced reparative angiogenesis in OIR model (Figs 4, 5). **B**. In response to hyperoxic-ischemic insult, deletion of p75^NTR^ decreased apoptotic signal, preserved survival signal by decreasing proNGF and enhancing NGF and TrkA expression and activation (Figs 2, 3, 8). **C**. In response to hypoxic-angiogenic phase, deletion of p75^NTR^ increased revascularization of central retina by maintaining VEGF/VEGFR2 activation and restoring balance between NGF and BDNF and their corresponding precursors; proNGF and proBDNF (Figs 6, 7**;** Suppl. Fig. 3). **D**. Vascular protection observed in p75^NTR−/−^ was blunted by inhibition of NGF survival receptor; TrkA receptor at p12 (Figs 8, 9). These findings support the notion that modulating p75^NTR^ exert vascular protection by two-fold mechanism: one by inhibiting proNGF/p75^NTR^ apoptotic signal and one by activating NGF/TrkA survival and angiogenic signal via the cross-talk between neurotrophin receptors; p75^NTR^ and TrkA.

Growing body of evidence support the role of neurons in regulating angiogenesis by secreting growth factors and guidance cues (reviewed in^32^). NGF and BDNF are members of the neurotrophin family that can exert neuroprotective, vascular protective and angiogenic effects. As adaptive response to overcome ischemia, NGF will be released, initially as a precursor, proNGF that normally get cleaved to its mature form^12^. We and others have shown that pro-oxidative milieu can impair maturation of NGF leading to accumulation of proNGF and activation of p75^NTR^ pathway in the diabetic retina and ischemic heart^10,11^. Here, we show that exposure of WT pups to hyperoxia, a well-documented condition of oxidative and nitrative stress^33,34^ triggered the accumulation of proNGF compared to normoxic-controls. It is interesting that the ratio between NGF to proNGF was significantly reduced in hyperoxia that coincided with upregulation of its receptor p75^NTR^ and vascular cell death at p12 in WT. The apoptotic action of p75^NTR^ receptor via forming a co-receptor with sortilin is well-documented in both neuro- and microvascular degeneration (reviewed in^15^. While there was no change in sortilin expression in response to OIR exposure or p75^NTR^ gene deletion, we observed upregulation of p75^NTR^ protein expression at p12, a time point for maximum central vascular death in WT retina. In contrast, a recent study using similar OIR model showed that expression of p75^NTR^ receptor did not change in response to hyperoxia (p8, p10 and p12), yet it showed significant increase in response to hypoxia at p14 and p17^14^. Nevertheless, our results came in agreement with reports showing increased expression of p75^NTR^ in ischemic retina models including ischemia induced by elevated intra-ocular pressure^35^, and in an inherited retinal degeneration model, Royal College of Surgeons rats, that was associated with progressive capillary dropout and subretinal neovascularization^36^.

Deletion of p75^NTR^ receptor significantly attenuated vascular cell death as assessed by attenuated capillary dropout area at p12 **(**Fig. 4**)**. Our group has previously shown that vaso-obliteration at p12 is associated with increased expression of apoptotic markers including; cleaved caspase-3 and PARP as well as decreased activation of Akt^25,37^. Deletion of p75^NTR^-attenuated vascular cell death coincided with preserved activation of the survival Akt pathway as well as attenuated expression of apoptotic markers, cleaved and total-PARP **(**Fig. 3**)**. Deletion of p75^NTR^ reduced pathological retinal neovascularization (tufts) and stimulated reparative angiogenesis evident by smaller central avascular area by p17 **(**Fig. 5**)**. These results lend further support to recent studies using molecular or pharmacological inhibitor of p75^NTR^ that show vascular protection in different models of ischemic retinopathy^14,38^. Modulating VEGF expression was proposed as a potential contributor to vascular protection observed in p75^NTR−/−^ mice pups^38^. As expected, hypoxia triggered significant increase in gene and protein expression of VEGF in WT-pups **(**Suppl. Figs 2A, 6A**)**. This corroborates with previous findings^22,23,38^. Deletion of p75^NTR^ did not reduce, but rather sustained hypoxia-induced VEGF expression and VEGFR2 activation **(**Fig. 6**)**. These results support prior findings that gene delivery of p75^NTR^ impaired neovascularization and blood flow recovery in diabetic mouse with hind limb ischemia through depression of VEGF^39^, however our results came in contrast to the results of another study by Le Moan N *et al*.^38^, which showed that genetic deletion of p75^NTR^ receptor resulted in decreased stabilization of HIF-1α and VEGF expression by p17 in OIR model^38^. Our results are further supported by prior work showing that treatments that sustain VEGF/VEGFR2 activation stimulate reparative angiogenesis in addition to preventing retinal neovascularization in OIR model^23^.

Exposure to relative hypoxia did not alter p75^NTR^ expression at p14 or p17, a time point of maximum retinal pathological neovascularization **(**Fig. 1**)**. Our results came in agreement with a prior study showing that hypoxia stimulates shedding of p75^NTR^ rather than modulating its expression^38^. Moreover, exposure to relative hypoxia resulted in significant increase in proNGF and proBDNF at p14 in WT. In agreement, vitreal samples from patients with proliferative diabetic retinopathy showed significantly low levels of mature NGF^11,28^ and BDNF^40,41^. Clinically, preterm infants who experienced proliferative retinopathy had decreased serum levels of BDNF compared to full-term^42,43^. Down regulation of neurotrophins; NGF, BDNF, NT-3 and GDNF was also reported in an ischemic rat retina model^44^. Deletion of p75^NTR^ receptor significantly decreased the levels of proNGF and preserved the level of mature NGF **(**Fig. 2**)** at p12, an effect that was sustained at p14 **(**Fig. 7**)**. Same results were also found for BDNF where, deletion of p75^NTR^ preserved the ratio of mature BDNF level to the level of the precursor form; proBDNF **(**Supplementary Fig. 3**)**. Our results lend further support to prior studies showing that silencing p75^NTR^ expression or genetic deletion of p75^NTR^ corroborates with restoring balance of NGF to proNGF ratio^13,45^, as well as balance of neurotrophin-3 (NT-3) to proNT-3 ratio^46^.

One of the cardinal findings of the current study is the observation that deletion of p75^NTR^ preserved the ratio between mature NGF and its precursor proNGF under both hyperoxic-ischemic phase and hypoxic-angiogenic phase **(**Figs 2, 7**)**. Restoring the balance of NGF/proNGF coincided with significant increases in expression as well as activation of TrkA receptor in p75^NTR−/−^ pups by p12 **(**Fig. 8**)**. The crosstalk between downplaying p75^NTR^ and enhancing TrkA signal was maintained throughout the neovascularization phase of OIR, however to lesser extent **(**Figs 7, 8**)**. The cross-talk between p75^NTR^ and TrkA has been demonstrated by several groups. We and others have shown that upregulation of p75^NTR^ expression concurred with inhibition of Trk-Y490 phosphorylation^26,27,47^. Treatments that enhanced TrkA-activation was associated with inverse effects on p75^NTR^ expression^26^ or activation^48^. The current findings lend further support to prior reports showing that downregulation of p75^NTR^ enhanced activation of TrkA pathway^13,18,45,49^. However, this is the first study that demonstrated shifting the balance by decreasing p75^NTR^ and increasing TrkA in retinal angiogenesis model. Previous literature identified clear angiogenic effects of NGF via activation of TrkA in models of angiogenesis^7,50^. Other studies identified proangiogenic effects of proNGF via activation of TrkA *in vitro* and *in vivo*^17,18,51^. Therefore, our results support the hypothesis that deletion of p75^NTR^ receptor preserved a balanced ratio of NGF/proNGF that can dictate the extent of TrkA survival and angiogenic signal and afford vascular protection. Next, we examined the impact of blocking TrkA receptor using compound K-252a, a general and potent inhibitor of Trk receptors^30^ that was delivered intravitreally at the end of ischemic phase at p12 **(**Fig. 9**)**. Treatment with K-252a blunted the protective effects including amelioration of central avascular area and retinal neovascularization observed during hypoxic stage of OIR in p75^NTR−/−^ pups. We believe that this is the first report that demonstrates direct involvement of TrkA to mediate vascular protection observed by genetic deletion of p75^NTR^ receptor in both ischemic and angiogenic phases of OIR model.

## Conclusion

Here, we demonstrate a number of unexplored pathways that unraveled novel pathways by which the genetic deletion of p75^NTR^ receptor can contribute to vascular protection including; preserved NGF and BDNF signal and sustained VEGF/VEGFR2 activation that together maintain healthy retinal vasculature. Deletion of p75^NTR^ can impact TrkA expression and activation, suggesting that the cross talk between these two receptors can be utilized not only to down regulate one of them but also to up regulate the other receptor endogenously. These findings support neurotrophins as viable therapeutic targets to combat retinal diseases with aberrant angiogenesis.

## Electronic supplementary material

Supplementary figure
