# Supplementary material for: Deletion of p75NTR prevents vaso-obliteration and retinal neovascularization via activation of Trk- A receptor in ischemic retinopathy model
Source: Sci Rep. 2018 Aug 21;8:12490. doi: 10.1038/s41598-018-30029-0 (PMC6104090; doi:10.1038/s41598-018-30029-0)
Supplement: Supplementary file 1 — Supplementary figure [file 41598_2018_30029_MOESM1_ESM.pdf]

Deletion of p75^{NTR} prevents vaso-obliteration and retinal neovascularization via activation of Trk A receptor in ischemic retinopathy model

Sally L. Elshaer and Azza B. El-Remessy

Supplementary Figure 1

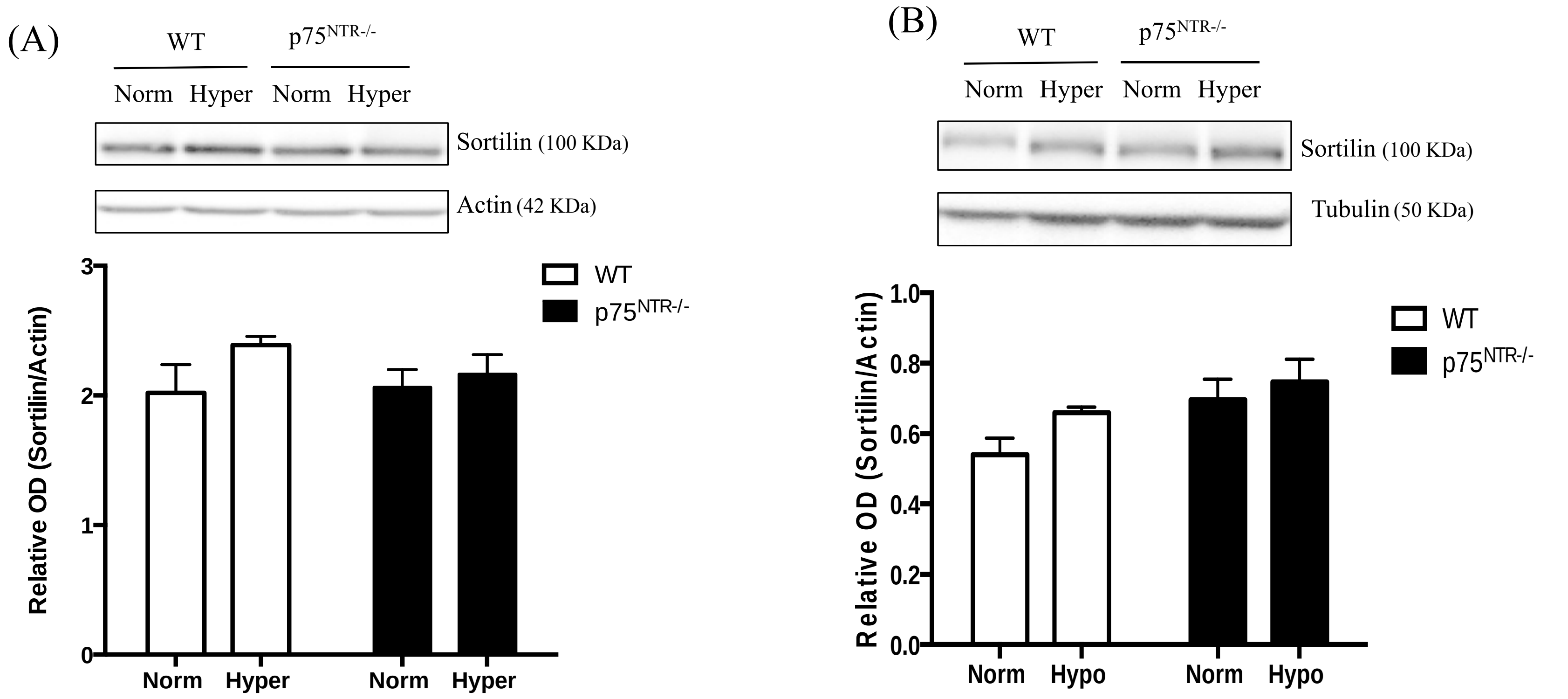

Supplementary Figure 1. Deletion of p75^{NTR} receptor did not alter sortilin receptor expression during vaso-obliteration and neo-vascularization stages of OIR. Representative Western blotting and bar graph analysis of sortilin expression in WT and p75$^{NTR-/-}$ pups during hyperoxic stage (A) and hypoxic stage (B) of OIR. Deletion of p75^{NTR} receptor did not significantly alter sortilin expression during both stages (n=4).

Deletion of $p75^{NTR}$ prevents vaso-obliteration and retinal neovascularization via activation of Trk A receptor in ischemic retinopathy model

Sally L. Elshaer and Azza B. El-Remessy

Supplementary Figure 2

(A) (B)

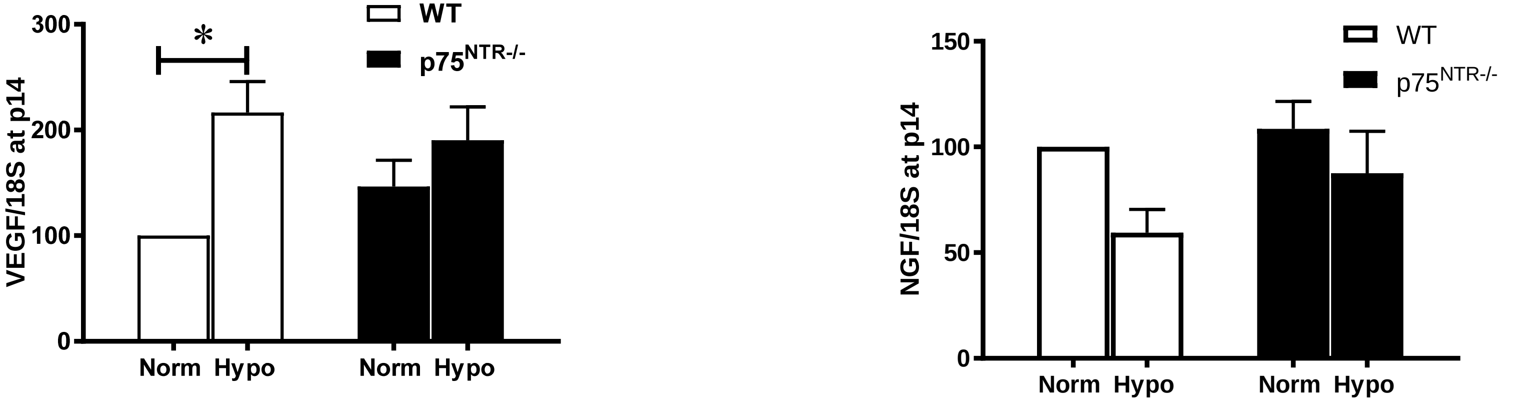

Supplementary Figure 2. Gene expression of VEGF and NGF during hypoxic stage of OIR in WT and $p75^{NTR-/-}$ pups. (A) Quantitative real-time PCR of VEGF gene expression in p14 WT and $p75^{NTR-/-}$ exposed to OIR. Two-way ANOVA showed overall significant impact of hypoxia in increasing VEGF expression. Post-hoc analysis showed significant effect of hypoxia to trigger VEGF expression in WT and a trend to increase but did not reach statistical significance in $p75^{NTR-/-}$ (*, $p<0.05$ versus normoxic control, n=6). (B) Quantitative real-time PCR of NGF gene expression in p14 WT and $p75^{NTR-/-}$ exposed to OIR. Two-way ANOVA showed overall significant impact of hypoxia in decreasing NGF expression, however post-hoc analysis did not reveal significant difference among various groups (*, $p<0.05$ versus normoxic control, n=4-5).

Deletion of p75^{NTR} prevents vaso-obliteration and retinal neovascularization via activation of Trk A receptor in ischemic retinopathy model

Sally L. Elshaer and Azza B. El-Remessy

Supplementary Figure 3

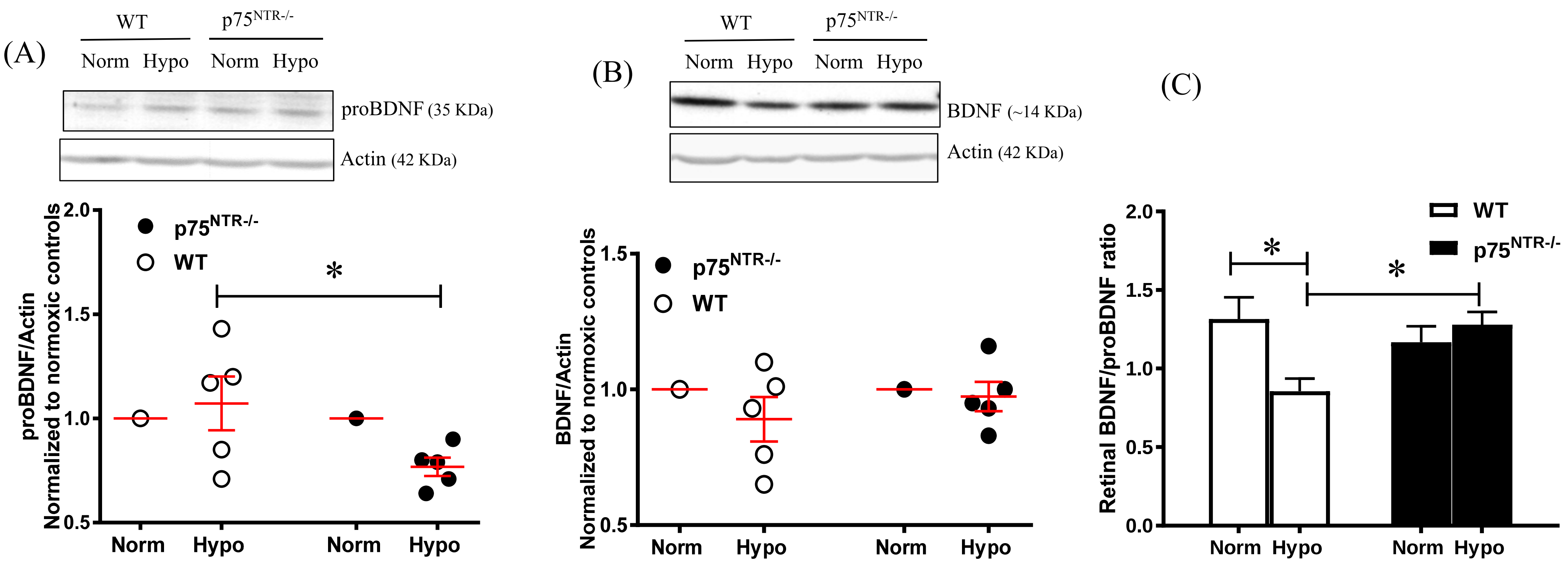

Supplementary Figure-3. Deletion of p75^{NTR} attenuated hypoxia-induced increase in proBDNF and restored ratio of BDNF/proBDNF. (A) Representative Western blotting and scatter graph for proBDNF by p14 showing that relative hypoxia significantly increased proBDNF level in WT-pups but not in p75$^{NTR-/-}$ pups (*, significant compared to WT-hypoxia group using two-way ANOVA, $p<0.05$, n=4). (B) Representative Western blotting and scatter graph for BDNF expression by p14 showing preserved level in p75$^{NTR-/-}$ pups during hypoxic stage of OIR. (C) Bar graph of BDNF/proBDNF ratio showing that hypoxia caused significant decrease in WT pups but not in p75$^{NTR-/-}$ pups (n=4-5). (*, significant using two-way ANOVA, $p<0.05$, n=4-5).

Deletion of p75^{NTR} prevents vaso-obliteration and retinal neovascularization via activation of Trk A receptor in ischemic retinopathy model

Sally L. Elshaer and Azza B. El-Remessy

Supplementary Figure 4

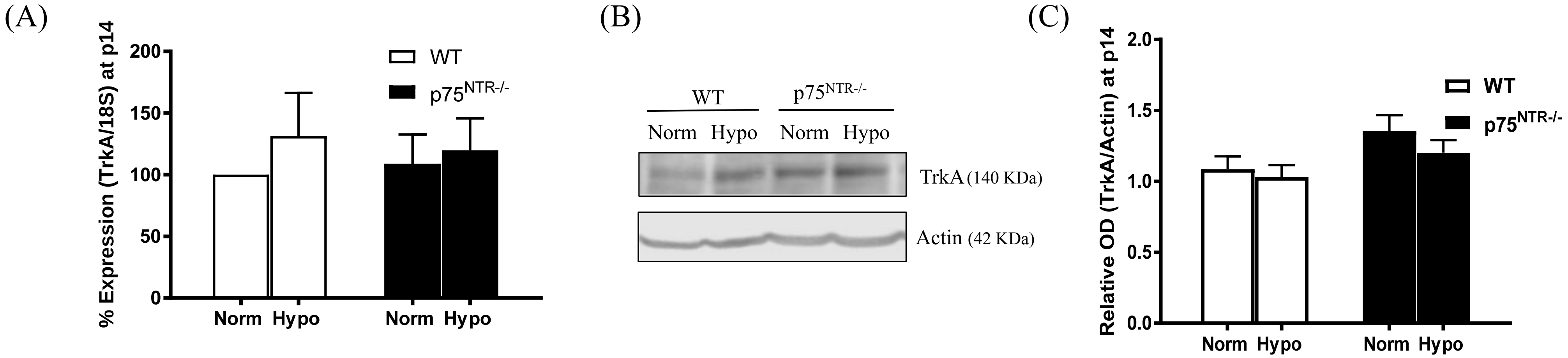

Supplementary Figure 4. Gene and protein expression of TrkA receptor during hypoxic stage of OIR in WT and p75$^{NTR-/-}$ pups. (A) Quantitative real-time PCR of TrkA gene expression in p14 WT and p75$^{NTR-/-}$ exposed to OIR showing no significant effect of hypoxia or p75^{NTR} deletion on TrkA gene expression (n=4). (B) Representative Western Blotting and bar graph analysis of TrkA protein expression at p14 showing no alteration by hypoxia or p75^{NTR} deletion (n=6-9).

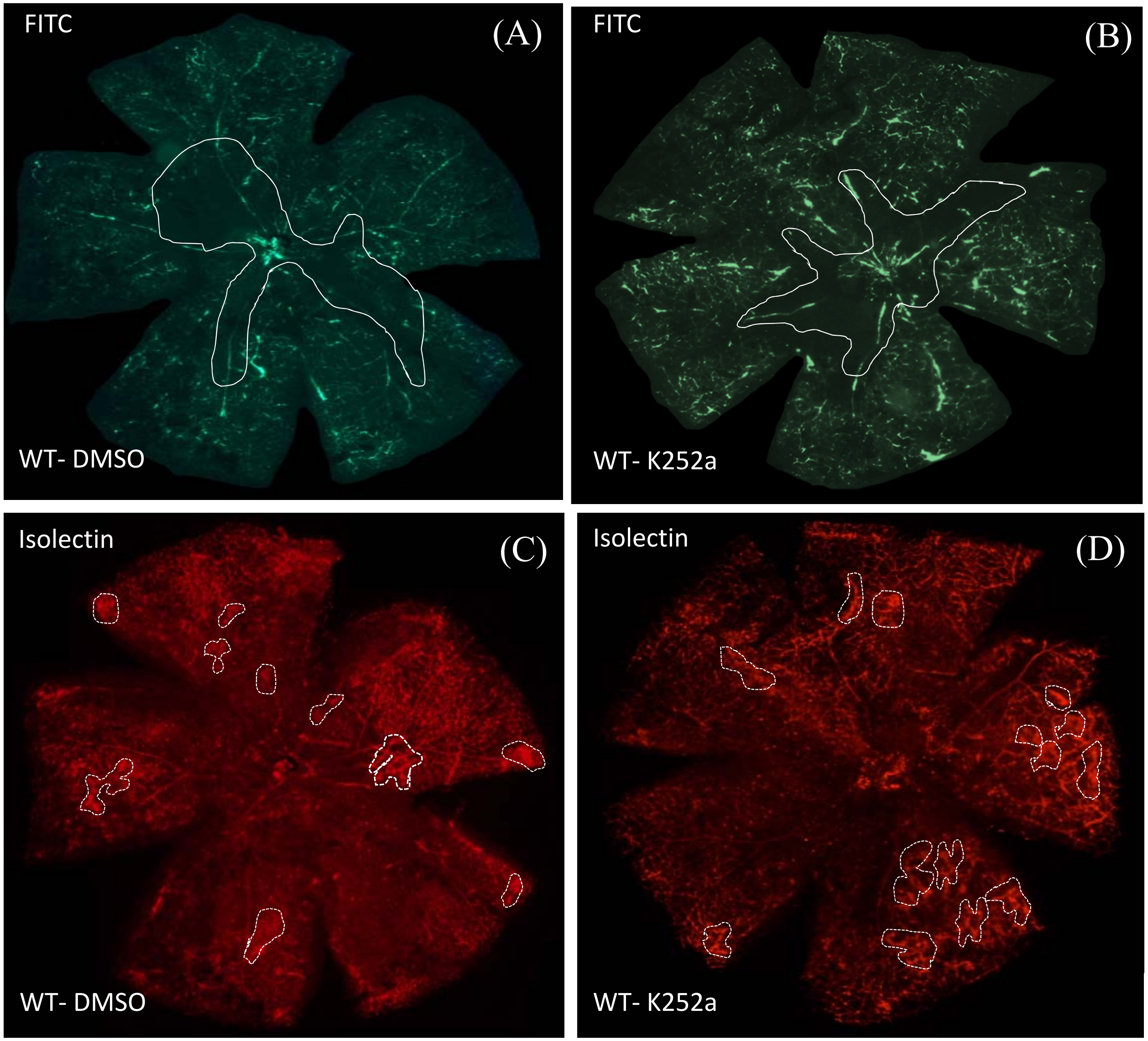

Deletion of p75^{NTR} prevents vaso-obliteration and retinal neovascularization via activation of Trk A receptor in ischemic retinopathy model

Sally L. Elshaer and Azza B. El-Remessy

Supplementary Figure 5. Inhibition of TrkA activity using K252a did not worsen retinal vascular injury in WT pups exposed to OIR. A-B. Representatives of p17 FITC-perfused retinal flat mounts of WT exposed to OIR and receiving DMSO or K252a (0.5µg µL^{-1} /eye) showing comparable central avascular area encircled in the center by white line, 5X magnification. **C-D.** Representatives of p17 Isolectin GS-stained retinal flat mounts of the same WT retinas exposed to OIR and receiving DMSO or K252a (0.5µg µL^{-1} /eye) showing comparable areas of mid-peripheral retinal neovascularization.
